# Neural modelling of the encoding of fast frequency modulation

**DOI:** 10.1371/journal.pcbi.1008787

**Published:** 2021-03-03

**Authors:** Alejandro Tabas, Katharina von Kriegstein

**Affiliations:** 1 Chair of Cognitive and Clinical Neuroscience, Faculty of Psychology, Technische Universität Dresden, Dresden, Saxony, Germany; 2 Max Planck Institute for Human Cognitive and Brain Sciences, Leipzig, Saxony, Germany; University of California at Berkeley, UNITED STATES

## Abstract

Frequency modulation (FM) is a basic constituent of vocalisation in many animals as well as in humans. In human speech, short rising and falling FM-sweeps of around 50 ms duration, called formant transitions, characterise individual speech sounds. There are two representations of FM in the ascending auditory pathway: a spectral representation, holding the instantaneous frequency of the stimuli; and a sweep representation, consisting of neurons that respond selectively to FM direction. To-date computational models use feedforward mechanisms to explain FM encoding. However, from neuroanatomy we know that there are massive feedback projections in the auditory pathway. Here, we found that a classical FM-sweep perceptual effect, the sweep pitch shift, cannot be explained by standard feedforward processing models. We hypothesised that the sweep pitch shift is caused by a predictive feedback mechanism. To test this hypothesis, we developed a novel model of FM encoding incorporating a predictive interaction between the sweep and the spectral representation. The model was designed to encode sweeps of the duration, modulation rate, and modulation shape of formant transitions. It fully accounted for experimental data that we acquired in a perceptual experiment with human participants as well as previously published experimental results. We also designed a new class of stimuli for a second perceptual experiment to further validate the model. Combined, our results indicate that predictive interaction between the frequency encoding and direction encoding neural representations plays an important role in the neural processing of FM. In the brain, this mechanism is likely to occur at early stages of the processing hierarchy.

## Author summary

Humans’ ability to understand and produce speech is one of the most fascinating developments of evolution. It is critical for smooth daily routines at the individual to the societal level. The computational mechanisms that the human brain uses for excelling at speech recognition are far from understood. One of the fundamental building blocks of speech are so-called formant transitions that characterise different speech sounds. To date, formant transitions are assumed to be processed according to a representational framework. In this view, the brain processes auditory signals in a hierarchical constructive way, where the higher levels of the hierarchy, that represent the formant transition directions, are informed by the neural representations of individual frequencies at the lower levels, but not vice versa. Here, we show that the representational framework does not fully explain human behaviour. Instead we develop a novel computational model in which the neural representations of formant transitions influence lower-level representations. This mechanism effectively increased the speed and efficiency of the recognition of formant transitions. The model explained previously unaccounted phenomena in human perceptual behaviour. These neural principles can be extended to other auditory processing networks and sensory modalities, and can be incorporated to neurobiologically-inspired automatic speech recognition algorithms.

## Introduction

Frequency modulation (FM) is a basic acoustic feature of animal vocalisation, human speech, and music. In human speech, consonants preceding and following a vowel can be acoustically characterised by formant transitions: a series of simultaneous fast FM sinusoids of around 50 ms duration that start or finish in the frequencies characterising the vowel [1]. At all stages of the ascending auditory pathway, FM is represented along the tonotopic axis in a *spectral representation* that encodes the instantaneous frequency of the stimuli [2]. Individual neurons at higher levels of the processing hierarchy (inferior colliculus [3–5], medial geniculate body [6, 7], and auditory cortex [8–11]) also encode FM direction and FM rate, by responding selectively to certain rates and direction. We call this latter, more abstract representation, the *sweep representation*.

Despite the massive feedback projections that characterise the auditory pathway [12, 13], computational models to date use only feedforward mechanisms to explain FM encoding [14]. Given the importance of high-order predictive elements in the optimisation of speech (e.g., [15]) and FM [16] recognition, descending projections are likely to play an important role for the encoding of fast FM-sweeps in the auditory system. However, to date there is no comprehensive model of such fast FM encoding that incorporates both, the sweep and the spectral representations, and describes the potential sweep-to-spectral feedback mechanisms active during the processing of FM sounds. The aim of the present study was to develop such a model, with a focus on FM-sweeps of the duration and frequency span of formant transitions.

To do that we harnessed a classical behavioural effect from psychoacoustics first reported around 60 years ago [17], which we will refer to as the sweep pitch shift. In the original experiment, participants listened to fast rising and falling FM-sweeps. The authors discovered that the participants judged up sweeps as eliciting a higher pitch than down sweeps with the same average fundamental frequency. These findings were later replicated [18, 19], although a reliable quantitative assessment of the phenomenon using stimuli with a controlled spectrum is lacking. To explain the effect d’Alessandro and colleagues proposed a phenomenological model assuming that the pitch of a sweep is integrated using a fixed-size window from the instantaneous frequency of the stimulus across time [20, 21]. Due to the leaking memory of the integration, this process naturally favours the latest frequencies of the sweep, explaining the sweep pitch shift. However, the authors found that different integration weights were necessary to explain different partitions of their data, indicating that the phenomenological model is not a parsimonious explanation of the sweep pitch shift.

Using the classical behavioural effect we approached the development of a comprehensive FM-encoding model in three steps. First, we re-examined and quantified the sweep pitch shift in a behavioural experiment, and tested whether the experimental data could be explained by existing computational models [22–24]. We found that mechanistic models of pitch processing that attempt to describe the circuitry underlying perception rather than perceptual phenomena [25] could not explain the sweep pitch shift. Current models of FM encoding [14] consider a static representation of spectral information, and thus they predict that the sweep pitch shift would not occur. Thus neither existing models of pitch processing nor FM-encoding could explain the sweep pitch shift. In a second step we built a hierarchical model motivated by the hypothesis that the sweep pitch shift results from the modulation exerted by feedback projections between the sweep and spectral representations. The feedforward components of the model were based on results of previous studies on FM direction selectivity and included processing of instantaneous frequency and processing of FM direction [10, 14, 26]. The feedback architecture was based on generative hierarchical models and predictive coding [27, 28] and informed by the human psychophysics results from the first part of the study. In the third and last step, we used a new set of stimuli termed *sweep trains* to further validate the model. These stimuli, consisting of a concatenation of five FM-sweeps, preserve the same acoustical features of the original FM-sweeps but elicited different dynamics in the feedback system of the model than their single-sweep counterpart. The ability of the model to predict the pitch elicited by these new stimuli illustrated that the feedback mechanisms proposed in this work, and not bottom-up acoustical features of the stimuli, are the driver of the sweep pitch shift.

## Results

### The sweep pitch shift revisited

First, we re-examined and quantified the sweep pitch shift, measured as the difference between the perceived pitch and the average frequency of the sweep: $\Delta p=f_{\text{perceived}}-\bar{f}$. Eight participants matched a total of 30 fast FM sweeps with frequency spans Δ*f* ∈ [−600, 600] Hz and 3 average frequencies $\bar{f}\in \{900,1200,1500\}\;\text{Hz}$ (see Methods). Each sweep had a duration of 40 ms and was preceded and followed by 5 ms of constant frequency. Participants’ task was to match each sweep to a pure tone, which frequency was used to determine the elicited pitch of the sweep.

**Fig 1:**
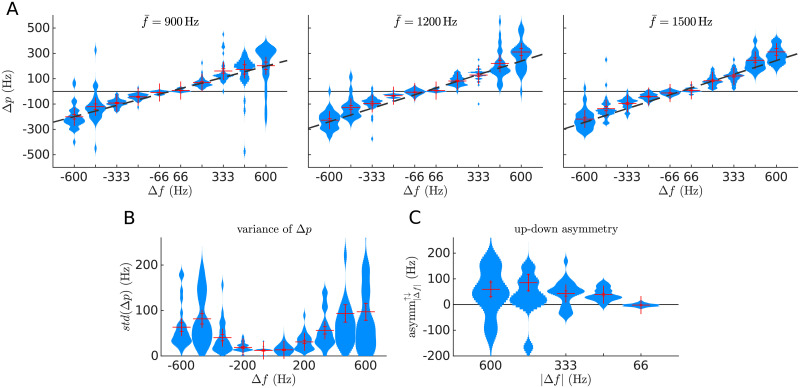
Sweep pitch shift. A) Kernel density estimations on the perceived pitch are plotted separately for each of the 30 FM-sweeps used in experiment 1; each of the three panels displays the data of each of the three average frequencies $\bar{f}$. Data points correspond to the average sweep pitch shift $\Delta =f_{\text{perceived}}-\bar{f}$ (*y*-axis) of a target sweep with a specific frequency span Δ*f* (*x*-axis) reported by one participant. Red crosses show the mean and standard error of the average Δ*p* across participants. Dark dashed lines show the linear fit of the average Δ*p* across participants. B) Kernel density estimations of the subjects’ standard deviation of the sweep pitch shift Δ*p*, plotted separately for the different frequency spans Δ*f*. Each sample in the distributions corresponds to the standard deviation of the perceived pitch of a sweep in one subject (i.e., in each distribution there are 8 × 3 points, one for each subject and $\bar{f}$). The standard deviation is monotonically correlated to the absolute span |Δ*f*| (Pearson’s *r* = 0.48, *p* < 10^−14^). C) Kernel density estimations of the up/down asymmetry asymm^↑↓^ distributions as defined in Eq (1). Each sample of the distributions corresponds to the difference of the average absolute deviation from centre frequency between up and down sweeps of the same |Δ*f*| for a given subject and centre frequency (*N* = 8 × 3 = 24). Red crosses show the mean and the standard error of the data.

The pitch shift Δ*p* depended on the sweep’s span Δ*f* (Fig 1A and S1 Table). The exact dependence was consistent across participants for sweeps with Δ*f* ≤ 333 Hz lying in the vicinity of the linear fit $f_{\text{perceived}}≃\bar{f}+m\;\Delta f$. There was an average deviance from the fit of 46 Hz. Sweeps with larger frequency spans resulted in wider distributions of *f*_perceived_ (Pearson’s *r* = 0.48, *p* < 10^−14^; Fig 1B); all subjects showed the same sweep pitch shift direction and comparable orders of magnitude on the dependence of Δ*p* with Δ*f* (S1 Fig). Presenting the sweep before or after the probe tone did not systematically affect the perceived pitch (S2 Fig). Raw data is available in an external repository (github.com/qtabs/fmPitch).

In their classical study, Brady and colleagues [17] showed that the absolute value of the sweep pitch shift |Δ*p*| is larger for down than for up sweeps. In a later study, Nabelek and colleagues [18] showed the reversed effect. To test if our data replicates any of these previous findings, or shows no up/down asymmetry at all, we drew, for each absolute frequency span |Δ*f*|, the distribution of the differences between the pitch shift in up and down sweeps:

$$\begin{array}{c} {\text{asymm}}_{\left|\Delta f\right|}^{↑↓}=\left|\Delta p\left(\Delta f\right)\right|-\left|\Delta p\left(-\Delta f\right)\right| \end{array}$$ (1)

Our results robustly replicated the observations from Nabelek and colleagues (Fig 1C). The sweep pitch shift was significantly larger for up than down sweeps for |Δ*f*| ≥ 200Hz (*p* < 2 × 10^−5^) but not for |Δ*f*| = 66Hz (*p* = 0.77), according to two-tailed rank-sum tests (number of samples *N* = 96).

Last, we tested if the dependence of the sweep pitch with Δ*f* was robustly replicated across subjects. The slopes of the linear fits between *f*_perceived_ and Δ*f* were of similar magnitude in all participants (mean slope *m* = 0.38, standard deviation across subjects *σ*_*m*_ = 0.07, a 18.5% of the nominal value, corresponding to an effect size of *d* = 5.4; see S1 Fig).

### Bottom-up models of pitch do not explain the sweep pitch shift

Current theories of pitch suggest that two complimentary codes of pitch coexist in the auditory system: the spectral or place code, produced by the spectral decomposition of the stimuli performed by the basilar membrane; and the temporal code, comprised in the spike timings of the neurons across the auditory pathway that are phase locked to the stimulus waveform (see [29] for a review). If the sweep pitch shift was a consequence of bottom-up pitch processing, we would expect the effect to be explainable by computational models that use either of the two representations to infer pitch. To test this we computed the pitch predicted by one representative model of each family; i.e., one model using the spectral and one model using the temporal codes. Although the possibility that the auditory system integrates information from both codes has been theoretised (e.g., [25]) and implemented [30] before, the combination of both codes has so far proposed to be purely additive. Thus, a combined spectro-temporal approach could only explain the sweep pitch shift if at least one of the two codes shows a positive pitch shift for up sweeps (Δ*f* > 0) and a negative pitch shift for down sweeps (Δ*f* < 0).

**Fig 2:**
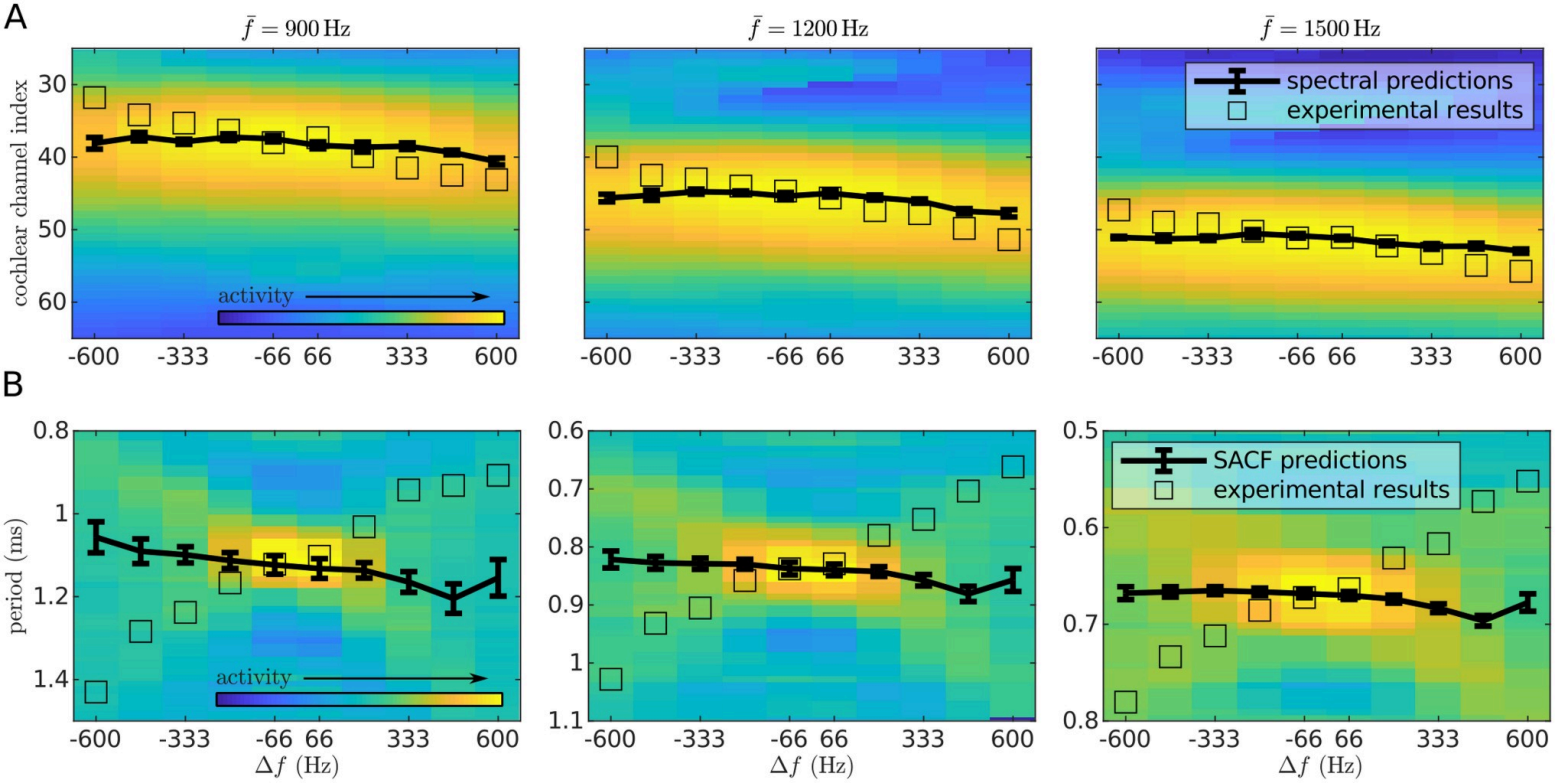
Predictions of the bottom-up models of pitch. A) Predictions of the spectral model. Heatmaps show the mean activation across the duration of the stimuli at different cochlear channels, as simulated by a model of the auditory periphery in response to each sweep; units are arbitrary. Error bars point to the expected value and standard deviation of the distribution across frequencies for each sweep. Each empty square denotes the expected channel elicited by a pure tone with the frequency of the average experimental data of the corresponding sweep. B) Predictions of the temporal model. Heatmaps show the distribution across periods elicited in the summary autocorrelation factor (SACF) for each sweep. The value corresponding to each period was computed as the average activation of the SACF across four harmonics (see Methods); heatmap units are arbitrary. Error bars point to the expected value and standard deviation of the distribution across periods for each sweep. Each empty square denotes the expected period elicited in the SACF by a pure tone with the frequency of the average experimental data of the corresponding sweep.

In the spectral model, pitch can be directly inferred by computing the expected value of the activity across cochlear channels in the auditory nerve [22, 25]. Predictions of the spectral model approximate the empirical data for Δ*f* ∼ 0. Unlike the empirical data, however, predictions of the spectral model show no systematic dependence of *f*_perceived_ on Δ*f* (Fig 2A). More sophisticated spectral models designed to explain how the pitch of harmonic complex tones is encoded [25] would yield identical results because the sinusoidal FM-sweeps used in the present experiment evoke a single peak in the spectral distribution.

The temporal model was based on the principles of the summary autocorrelation function (SACF), that measures pitch according to the phase-locked response in the auditory pathway [23, 31]. We chose this model because it performs a relatively straightforward analysis of phase-locked activity. As in the spectral model, predictions of the SACF approximate the empirical data for Δ*f* ∼ 0, but do not show the dependence with Δ*f* observed in the data (Fig 2B). This is most likely a consequence of the 2.5 ms integration time window for phase-locked activity in the auditory system [32] being too large to integrate the rapidly changing frequencies of our stimuli (up to 15 Hz/ms).

We selected these two representative models of pitch processing because they keep the largest possible amount of information from the peripheral system. Our reasoning is that, if the sweep pitch shift cannot be derived from a minimally-processed code extracted from the peripheral output, it is very unlikely that it can be derived from any further bottom-up processing of this information.

### The FM-feedback spectral model

**Fig 3:**
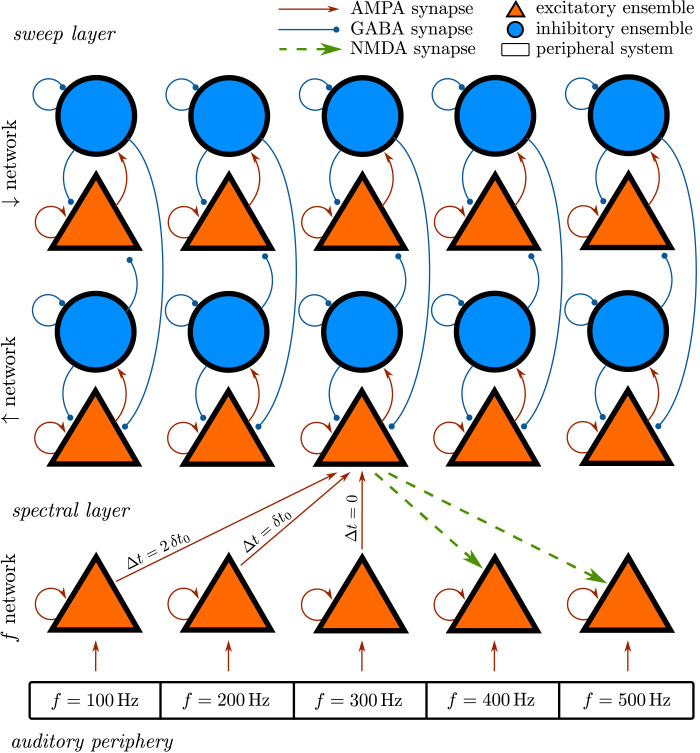
Diagram of the FM-feedback spectral model. The model consists of two levels: first, the *spectral layer*, with a network integrating the spectral information of the sound (*f* network); and second, the *sweep layer*, with one network specialised in detecting up (↑ network) sweeps and another network specialised in detecting down (↓ network) sweeps. The spectral layer integrates afferent inputs from the auditory periphery and encodes a representation of the stimulus that can be used to infer pitch. The sweep layer receives afferent inputs from the spectral layer that are used to decode the direction of the sweeps. Feedback connections from the sweep layer to the spectral layer modulate the time constants of the populations that are expected to be activated once the direction of the sweep has been decoded. The inhibitory ensembles in the up and down network enforce competition between up and down ensembles in a winner-take-all fashion. Note that the diagram is schematic and shows only 5 of the *N* = 100 populations and a single example of the connections between the sweep and the spectral layers. The labels of the boxes of the peripheral system are also schematic: the spectral resolution of the peripheral system is much higher. See Methods for specific details on the mathematical formulation of the model.

In this section we introduce a hierarchical model of FM-encoding in the auditory system, termed *FM-feedback spectral model*, with two levels (Fig 3). In the first level, the *spectral* layer holds a spectral representation of the sound. In the second level, the *sweep* layer encodes FM-sweep direction. The spectral layer uses the spectral rather than the temporal code to represent the instantaneous frequency of the stimuli because the integration window of the auditory system is too large to integrate the rapidly changing frequencies of the sweeps used in our experiment [32]. The animal electrophysiology literature also converges in the notion that sweep direction and rate are decoded from the spectral, and not the temporal representation of the sounds [4, 6, 7, 11, 14, 33].

The main hypothesis introduced in the FM-feedback spectral model is that, once the direction of the sweep is encoded in the sweep layer, a feedback mechanism modulates the effective time constant of the populations encoding the frequencies that are expected to be activated in the next instant in the spectral layer. We expect this mechanism to qualitatively explain why the posterior parts of the sweep are given a higher weight during perceptual integration and to quantitatively reproduce the exact dependence of pitch with Δ*f* observed in our data. An implementation of the FM-feedback spectral model written in python is freely available at github.com/qtabs/fmPitch.

**Fig 4:**
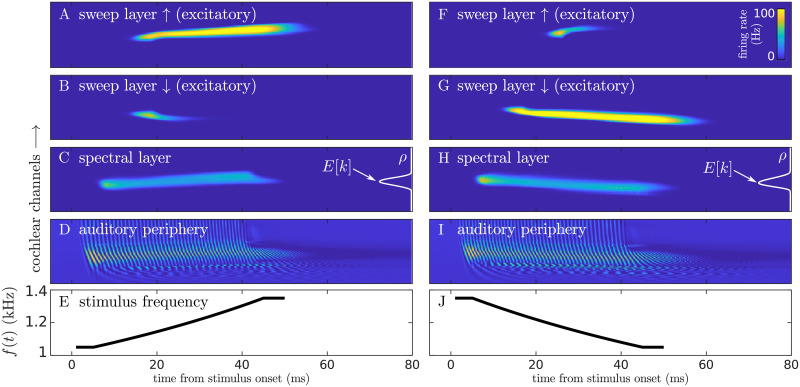
Model responses to an up and a down sweep. A-E show the responses to an up sweep, and F-J to a down sweep. From top to bottom: (A/F) the instantaneous predicted firing rate of the up-selective excitatory populations in the sweep layer; (B/G) the instantaneous firing rate of the down-selective excitatory populations in the sweep layer; (C/H) the instantaneous firing rate of the populations in the spectral layer and, in the right of the panels, a schematic view of the probability distribution of pitch derived from this representation; (D/I) the output of the model of the auditory periphery; (E/F) the instantaneous frequency of the sweeps along time. In all panels except for E/J, *y*-axis represents the cochlear channel *n*, ordered from bottom (lowest best frequency) to top (highest best frequency). The stimuli were the up and a down sweeps with Δ = ±300Hz and $\bar{f}=1200\;\text{Hz}$ used in the experiment.

Example responses of the excitatory populations of the model to up and down sweeps are shown in Fig 4.

#### Modelling FM direction selectivity

We modelled FM direction selectivity using the principles of delayed excitation, a mechanism where neurons with different best frequencies output to the direction selective neuron with different delays [6, 10, 14, 26]. This mechanism introduced consistent delays between the populations in the spectral and the sweep layers. A sweep population receiving direct input from the spectral population encoding *f*_0_ and responding selectively to up sweeps will receive increasingly delayed inputs from the spectral populations centred at *f* < *f*_0_ (Fig 3). The relative delay in the connection between a spectral population *m* and a target sweep population *n* depends linearly on the spectral distance between the two ensembles: *δt*_*nm*_ = |*n* − *m*|*δt*_0_. Although this configuration is optimal for linear sweeps with slopes ≃ *δt*_0_/(*f*_*n*_ − *f*_*n*−1_), adding parallel replicas of these populations with varying *δt*_0_ would suffice to generalise the mechanism to a wider range of speeds and to non-linear sweeps. Populations selectively responding to specific rates have been reported in bats [34–37] and rodents [3, 8, 9].

The sweep layer consists of two networks, each encoding one of the FM directions and responding selectively to *up* (↑) and *down* (↓) sweeps. Each of the networks consists of *N* columns, each comprising an excitatory and an inhibitory population (Fig 3). Note that populations in the sweep network also have a best-frequency and they are thus arranged according to their corresponding cochlear channel: an up population in the sweep network responds selectively to up sweeps when these span through a certain frequency range (see Fig 4B).

**Fig 5:**
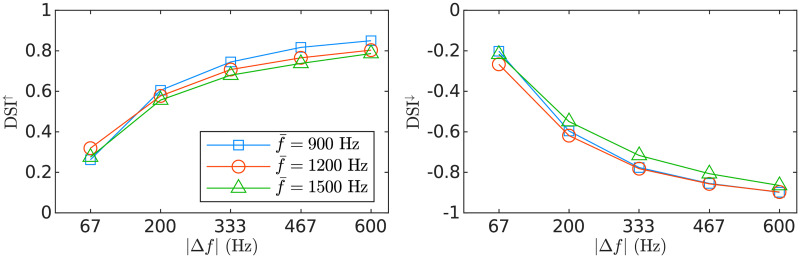
Direction selectivity indices for the FM-sweeps of the experiment. DSI^↑^ and DSI^↓^ to sweeps with different $\bar{f}$ and |Δ*f*|. DSI is defined as the proportional activity to up in comparison to down sweeps in a given network.

To quantify direction selectivity, we used the standard direction selectivity index (DSI; e.g., [11]), defined as the proportion of the activity elicited in a network by an up sweep minus the activity elicited in the same network by a down sweep with the same duration and frequency span. An ideal network responding selectively to up sweeps will have a DSI = + 1 and an ideal network responding selectively to down sweeps will have a DSI = −1. Similar DSI magnitudes are measured in the down and the up network (Fig 5). Network selectivity to FM direction was robust to variations of around 20% of the fitted value of the main parameters of the model pertaining direction selectivity (*δt*_0_, and the conductivities and dispersion in the connectivity matrices of the bottom-up connections). Deactivation of the feedback connections, however, resulted in a (16 ± 1.4)% average decrease in absolute DSI, indicating that the feedback connections sharpened direction selectivity.

Although we did not attempt to model FM rate selectivity, the DSIs monotonically increased with |Δ*f*| (Fig 5), a property that could be exploited in further developments of the model to encode modulation rate [3, 7, 9].

#### Predictive mechanisms

Once neurons in the sweep layer encoded the sweep direction, feedback connections targeting the spectral layer applied currents that facilitated the encoding of expected frequencies. We will call them facilitation currents in the following. Let *i* be the population in the up-sweep network receiving inputs from a population in the spectral layer encoding a certain frequency *f*_0_. Due to delayed excitation, the population *i* becomes active when it detects an up sweep occurring in the neighbourhood of frequencies *f* ≤ *f*_0_. Although when *f*_0_ is the ending frequency of the sweep the following frequencies will not activate next, most often *f*_0_ will be just an intermediate frequency within the sweep. Thus, activation of *i* would imply that populations in the spectral layer with best frequencies immediately higher than *f*_0_ are likely to activate next. The facilitation currents, encoded in the feedback projections stemming from the sweep layer and targeting the spectral layer, reduce the reaction time of the populations in the spectral layer that are expected to activate next using low-current feedback excitatory signals. Similarly, feedback connections stemming from a population *j* in the down-network that received timely inputs from a spectral population with best frequency *f* will target populations in the spectral network with best frequencies immediately lower than *f*_0_.

NMDA receptors are typically responsible for conveying feedback excitatory information in the cerebral cortex [38, 39]; specifically, NMDA-deactivation results in a reduced feedback control in the auditory pathway [40]. Thus, while bottom-up drive was modelled using AMPA dynamics, feedback connections were modelled according to NMDA-like synaptic gating dynamics with a finite rising time constant [41]. Feedback current intensity was kept low in comparison to the bottom-up driver by enforcing NMDA conductivity to be much smaller than the AMPA conductivity (i.e., *J*^NMDA^ ≪ *J*^AMPA^).

**Fig 6:**
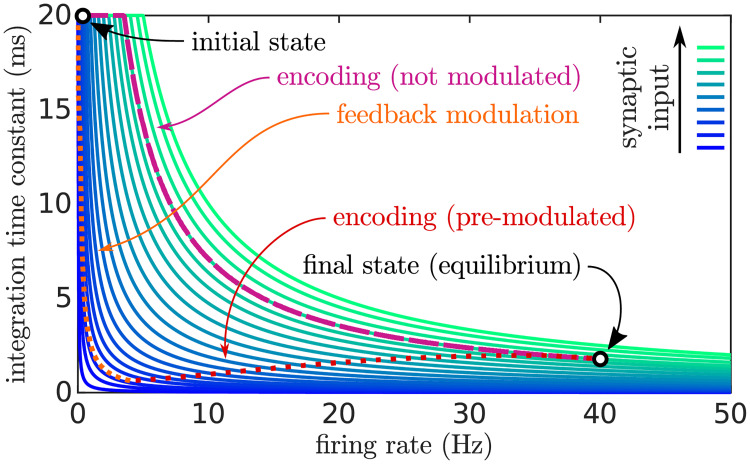
Effect of the predictive feedback mechanism on the population time constants in the spectral layer. *τ*^pop^(*h*, *I*) (green-blue solid lines) depends on the firing rate *h* and the synaptic input *I* (see Methods). The figure portraits two different trajectories of the variable *τ*^pop^(*h*, *I*) in the (*h*, *I*) space, both starting at an initial state (*h* ∼ 0 in the regime of spontaneous activity with no inputs) and finishing at an equilibrium state (with *h* ∼ 40 Hz). The dashed purple line shows a trajectory followed by the population when the forward synaptic input from the peripheral layer is plugged in without feedback modulation. In this case, the population reacts slowly to the strong synaptic input, and eventually converges to equilibrium. The dotted lines (orange and red) show the trajectory of the same population in the presence of feedback modulation (i.e., the facilitation currents). The low-current feedback excitatory signals drive the population to a state with a low effective time constant without substantially increasing its firing rate (orange section of the trajectory). When the strong synaptic input from the auditory periphery is switched on (red section of the trajectory) the population reacts quickly to the synaptic input, reaching equilibrium much faster than in the non-modulated case.

The facilitation currents modulated the spectral population that is expected to fire next so that it subtly increased its firing rate with respect to a not modulated population. Due to network effects captured in the mean-field model [42], this subtle activation driven by a low-current effectively reduces the neural population’s decaying time constant *τ*^pop^ (Fig 6), equivalent to a smaller integration time window of a leaky integrator. Endowed with a smaller effective integration time constant, the population integrates the sensory input faster and spends more time in the high-firing-rate regime than a population that has not been facilitated. Since facilitated populations spend more time in the high-firing-rate regime, frequencies expressed in the last part of the sweep have stronger contributions to the probability distribution of pitch.

Stimuli with constant frequency (e.g., pure tones) do not drive any of the sweep populations and thus do not activate any feedback mechanisms. Therefore, in the absence of FM, the model reduces to a purely bottom-up spectral model.

#### Reproduction of the sweep pitch shift by the FM-feedback spectral model

**Fig 7:**
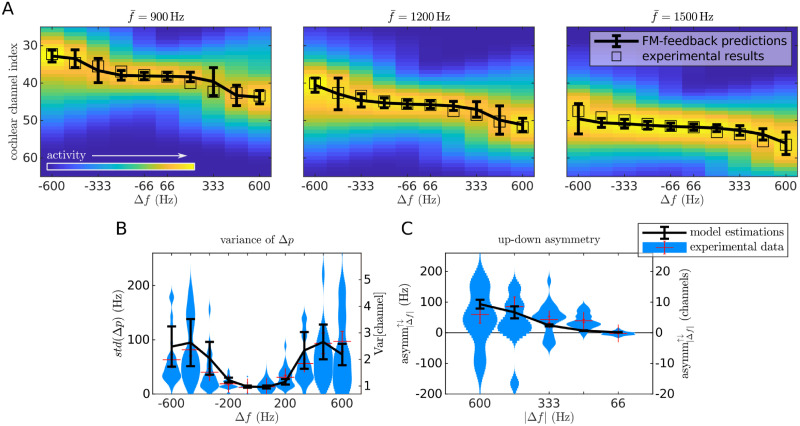
Predictions of the FM-feedback spectral model for FM-sweeps. A) Heatmaps show the mean activation at different cochlear channels as simulated by a model of the auditory periphery in response to each sweep; units are arbitrary. Error bars point to the expected value and standard deviation of the distribution across frequencies for each sweep. Each empty square denotes the expected channel elicited by a pure tone with the frequency of the average experimental data of the corresponding sweep. B) Predictions of the standard deviation of the perceived pitch. Error bars are estimations of standard error of the model calculated based on the dispersion of the centroids for different $\bar{f}$ and the standard deviations of the spectral distribution *ρ* of each condition. Kernel density estimations are the same experimental data as in Fig 1B. C) Predictions of the standard deviation and up/down asymmetry. Error bars show the model predictions of the up/down asymmetry coefficient asymm^↑↓^ (see Eq (1)) in the channel space. Kernel density estimations are the same experimental data as in Fig 1C.

The FM-feedback spectral model explains *R*^2^ = 0.97 of the variance of the experimental data (Fig 7A). Moreover, there was a significant correlation between the variance of the model responses and the standard error of the experimental data (*r*_*p*_ = 0.63, *p* < 10^−10^), indicating that the larger variability in the sweep pitch shift observed for the larger Δ*f* can be understood as a consequence of a wider spread activation across the spectral populations.

Up sweeps partially compensate for the differential delay in the basilar membrane responses to low frequencies with respect to high frequencies, provoking higher synchronisation in the auditory nerve [43]. Stronger peak activities result on slightly higher facilitation currents for up than for down sweeps, causing a noticeable stronger absolute mean pitch shift for up than for down sweeps, as observed in the experimental data (Fig 7B). Note that this is not the result of the model overfitting the data, since the average error of the model fit (*E*[error] ≃ 1 channel ≃ 50 Hz) is of the same order of magnitude as the up-down asymmetry *E*[asymm^↑↓^] ≃ 100 Hz.

**Fig 8:**
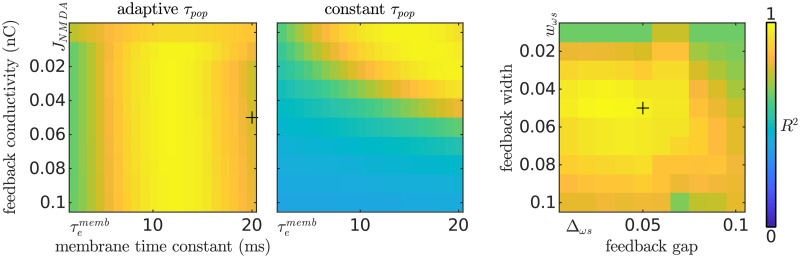
Experimental fit in relation to the FM-feedback spectral model parametrisation. Heatmaps show the explained variance of the experimental data *R*^2^. Unless stated otherwise, parameters not varied in the matrices correspond to the values listed in Table 1. The two leftmost plots show the dependence of *R*^2^ with the conductivity of the feedback connections and the dynamics of the excitatory population time constants. Different values of the nominal population’s time constant were used to illustrate that the dynamic effect (rather than the resulting shorter time constant) is crucial to explain the experimental results; however, during the parameter tuning the temporal constant was constrained to *τ*^memb^ = 20 ms based on physiological observations [44]. The rightmost plot shows the dependence of *R*^2^ on the width and reach (*w*_*ωs*_ and Δ_*ωs*_, respectively; see Methods) of the feedback connections. Black crosses in the parameter space signal the final parametrisation.

To study the dependence of the model fit with the model’s parameters we recomputed the explained variance *R*^2^ across the parameter space of the model (Fig 8). The model explained the experimental data in a wide section of the parameter space, with an average *R*^2^ across a 5-point diameter sphere around the final parameters of *E*[*R*^2^] = 0.88 ± 0.01. To show that the fit of the model was not simply caused by an overall stronger activation provoked by the facilitation currents, but by a decrease in the effective time constant of the populations, we also computed the dependence of *R*^2^ with the conductivity of the feedback *J*^NMDA^ while keeping the population time constant *τ* fixed to *τ* = *τ*^memb^ (see Methods). Even considering lower *τ*^memb^ than the physiologically valid nominal value *τ*^memb^ = 20 ms, without an adaptive *τ*, much stronger NMDA currents (*J*^NMDA^ ∼ *J*^AMPA^) are necessary to shift the peak of the distribution of the responses across the spectral layer towards the experimental results.

#### Reproduction of previous experimental results

**Fig 9:**
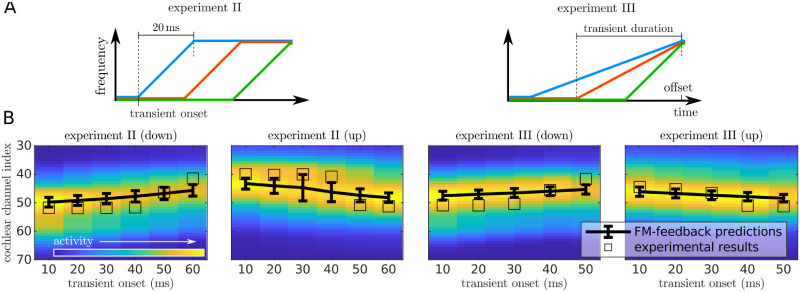
Predictions of the FM-feedback spectral model for Brady’s stimuli. A) Schematic view of the stimuli from [17]. In experiment II (left) the transient was fixed to a 20 ms duration and its onset was systematically varied so that the transition falls at different segments of the stimulus. In experiment III (right) the stimulus offset was fixed at 90 ms and the transient’s onset varied between 10 and 50 ms, resulting in transients of different durations. We extended the duration of these last stimuli to 95 ms to prevent the ramping at the end of the stimulus from overlapping with the FM transient. B) Model predictions. Heatmaps show the distribution of the activation across channels (*y*-axis) for different transient onsets (*x*-axis). Squares printed over the distributions mark the estimations of the experimental results in the channel space.

We tested whether the FM-feedback spectral model was able to predict the pitch shift of additional data. For this, we chose the stimuli of Brady and colleagues [17], because this was the only study that investigated the dependence of the sweep pitch shift with properties different than Δ*f*. Specifically, in the *experiment II* they considered FM-sweeps with a fixed 20 ms transition between 1000 Hz and 1500 Hz that was located at six different positions within a 90 ms stimulus (see schematics in Fig 9A, left). In the *experiment III*, they used FM-sweeps in the same Δ*f* but with transitions of six different durations (see schematics in Fig 9A, right). All stimuli had the same duration (90 ms) and frequency span (1000-1500 Hz); in each of the two experiments there was a total of 12 stimuli (six up, six down).

We compared the predictions of the FM-feedback spectral model with the experimental results reported in the original paper (Fig 9B). The experimental trend is well reproduced by the model (*R*^2^ = 0.49).

### Testing the FM-feedback spectral model with a new class of stimuli

The results described so far are in favour of the hypothesis that there is a feedback system between populations of the spectral and sweep representations that has strong repercussions on perceptual behaviour. Next, to validate these findings, we introduced a new set of stimuli specifically designed to contest the main hypothesis of the model. The new stimuli consist of concatenations of several single sweeps with the same properties as the stimuli used in the first experiment. We call them *sweep trains* in the following. Sweep trains present the same acoustical properties as the single sweeps used in the first behavioural experiment and should nominally elicit the same pitch percept as their single-sweep subcomponents. However, the FM-feedback spectral model predicts that the feedback system will only reduce the time constant of the spectral populations during the processing of the first sweep in the train, because they will already have an elevated firing rate (and thus a low effective time constant) during the processing of the subsequent sweeps in the train. Consequently, the model predicts that the sweep trains will elicit a much more subtle pitch shift than their single sweep counterparts. We tested this prediction in a perceptual experiment analogous to the first experiment.

### Sweep trains show minimal sweep pitch shift

**Fig 10:**
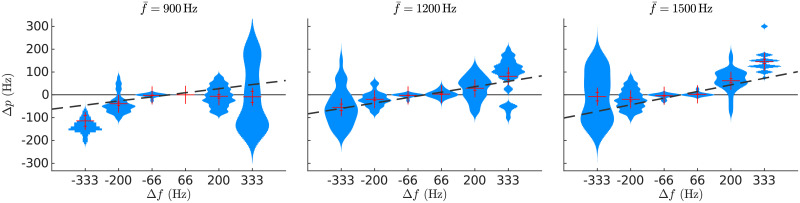
Sweep pitch shift for sweep trains. Kernel density estimations on the perceived pitch are plotted separately for each of the 18 sweep trains used in the experiment. The *y*-axis of each plot shows the magnitude of the sweep pitch shift Δ*p*. The x-axis list the spans of each of the sweeps. Red crosses show the mean and standard error of the data. Dark dashed lines show the group linear fit of the data.

Sweep trains were constructed using the sweeps from experiment 1. To ensure that each train was perceived as a single auditory object, we only used sweeps with |Δ*f*| ≤ 333 Hz, resulting in a total of 3 × 6 = 18 stimuli. As in the results from Experiment 1 (Fig 1), the magnitude of the pitch shift in sweep trains depended on Δ*f* (Fig 10 and S1 Table, bottom). However, as qualitatively predicted by the FM-feedback spectral model, the effect sizes of the correlation were lower than in the single-sweep experiment (cf., S1 Table, top). Data also showed higher variability than in experiment 1 (S1 Fig). After completing the experiment, some participants reported in informal conversation that the sweep train stimuli were harder to match than the single-sweep counterparts. Although trains with small Δ*f* were generally perceived as continuous tones, subjects reported that a few trains (putatively those with the largest Δ*f*) elicited a ringing-phone-like percept. Stimuli are available in the supporting information (S1 Sounds).

Sweep-train stimuli show only a subtle up/down asymmetry that did not reach statistical significance even for the larger Δ*f* (*p* = 0.67, *p* = 0.96, *p* = 0.36 for |Δ*f*| = 333, |Δ*f*| = 200, |Δ*f*| = 66, respectively; according to two-sided Wilcoxon signed rank tests with 24 samples per condition).

### The FM-feedback spectral model explains the diminished pitch shift in the sweep trains

**Fig 11:**
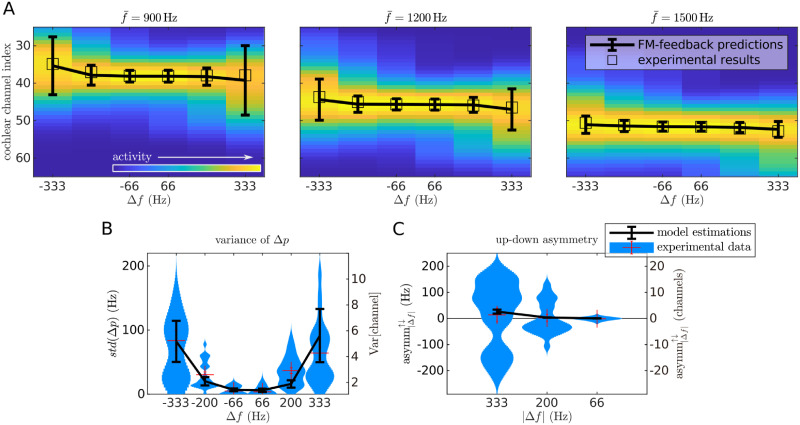
Predictions of the FM-feedback spectral model for sweep trains. A) Heatmaps show the distribution of the activation across the spectral representation (*y*-axis are cochlear channels) for different sweep frequency spans Δ*f* (*x*-axis). Error bars point to the expected value and standard deviation of the distribution across frequencies for each sweep. Each empty square denotes the expected channel elicited by a pure tone with the frequency of the average experimental data of the corresponding sweep. B) Predictions of the standard deviation of the perceived pitch. Error bars are estimations of standard error of the model calculated based on the dispersion of the centroids for different $\bar{f}$ and the standard deviations of the spectral distribution *ρ* of each condition. Kernel density estimations show the empirical subjects’ standard deviation of the sweep pitch shift Δ*p* in sweep trains, plotted separately for the different frequency spans Δ*f*. Each sample in the distributions corresponds to the standard deviation of the perceived pitch of a sweep in one subject. The standard deviation is monotonically correlated to the absolute span |Δ*f*| (*r*_*s*_ = 0.63, *p* < 10^−27^). C) Predictions of the standard deviation and up/down asymmetry. Error bars show the model predictions of the up/down asymmetry coefficient asymm^↑↓^ (see Eq (1)) in the channel space. Kernel density estimations show the empirical up/down asymmetry distributions in sweep trains, as defined in Eq (1). Each sample of the distributions corresponds to the difference of the average absolute deviation from centre frequency between up and down sweeps of the same |Δ*f*| for a given subject and centre frequency. Red crosses show the mean and the standard error of the data.

Next, we assessed the ability of the FM-feedback spectral model to quantitatively explain the effect size of the pitch shift observed in the sweep trains. The fit with the experimental data was comparable to that of the single sweep stimuli: the model explained *R*^2^ = 0.99 of the variance of the data (Fig 11A). As in the first experiment, the standard deviations of the experimental data was strongly correlated to the width of the model responses (*r*_*p*_ = 0.75, *p* < 10^−10^; Fig 11B).

Last, we tested whether the different up/down asymmetry (asymm^↑↓^) observed in the single sweeps and sweep train data could be quantitatively explained by the FM-feedback spectral model. In the single-sweep data, the model predicts a stronger sweep pitch shift magnitude |Δ*p*| for up sweeps (Fig 7C) because these elicit a stronger peak activation in the auditory nerve [45], resulting in stronger facilitation currents. Qualitatively, a much weaker asymmetry was expected in the sweep-train data, since the spectral populations have already high firing rates (and thus low effective integration time constants) during the processing of the ending four fifths of the stimuli. In sweep trains, then, only the first sweep contributes to the sweep pitch shift, whereas the remaining sweeps provide equal contributions to the range of spanned frequencies, diluting the shift magnitude by four fifths. Modelling results on the up/down asymmetry closely reproduced the empirical data (Fig 11C), fully explaining the observed differences between the two classes of stimuli.

## Discussion

In this work we have built a novel model that describes how feedback projections between the two different known representations of FM (i.e., spectral and sweep) could be used in the brain to facilitate encoding. This contrasts with the classical view of FM encoding as a bottom-up process [14]. The feedback mechanism proposed in this work uses predictions generated by populations encoding FM direction to aid encoding in populations encoding instantaneous frequency, enhancing direction selectivity and shortening FM processing time. Since this predictive facilitation is not intrinsically restricted to the fast-FM characteristic of formant transitions in speech, similar facilitation mechanisms could also boost encoding efficiency for the slower FM underlying the perception of prosody and melody.

In this work we have used the model to encode sinusoidal (pure-tone) FM stimuli that are far from the complexity of natural speech sounds. In speech, phonemes are characterised by concurrent formant transitions that span complementary frequency ranges. Moreover, each of these formant transitions are carried by harmonic complex tones rather than pure tones. It is currently unclear how the mechanisms introduced here could be used in natural settings to encode phonemes. One possibility is that sinusoidal sweeps are first decoded in the sweep layer and integrated in a later step of the ascending hierarchy. Since the populations encoding direction selectivity in the FM-feedback spectral model are tuned to specific best frequencies, the sweep layer is potentially able to encode simultaneous sweeps spanning complementary frequency ranges and represented by parallel harmonic series. Neural populations in the sweep layer could output to a third level of abstraction where combinations of concurrent sweeps across harmonic series are mapped into phonemes. Therefore, the FM-feedback spectral model could be the first basic building block towards a more comprehensive understanding of speech processing.

### Bottom-up pitch models and pitch codes

The bottom-up integration of the spectral representation, cornerstone of the classical spectral or place theories of pitch [46], predicted a null sweep pitch shift. Other attempts of bottom-up models have also failed to parsimoniously explain the pitch shift: A previous phenomenological model suggested that a leaky integration of the instantaneous frequency could result in the ending segments of the sweeps having a stronger weight in the pitch decision, which would qualitatively explain the direction of the pitch sweep shift [21]. However, this model predicts the same pitch shift in single sweeps than sweep trains and the same absolute pitch shift in up and down sweeps, in direct contradiction with the empirical data.

Our simulations showed that current bottom-up modelling approaches based on a temporal code cannot even extract robust pitch information from most FM-sweeps used in the experiments. This is most likely a consequence of the fast change rate in the periodicities of fast FM stimuli. Typically, pitch decisions based on the auditory nerve temporal code are made after integrating over four cycles of the period of the stimuli [32, 47], coinciding with the duration threshold for accurate pitch discrimination [48]. However, our stimuli presented an average change of ∼25 Hz across four repetitions of their average frequency, making this integration virtually impossible.

Another possibility is that a combination of the temporal and spectral codes is used to process the pitch of FM-sweeps, and that the sweep pitch shift emerges from this integration. Both, spectral and temporal representations of pitch play different roles in pitch processing, and it has been previously suggested that both codes could be added to perform pitch decisions (see [25] for a review). However, the temporal code had no usable pitch information at |Δ*p*| > 200 Hz that could be integrated with the spectral code.

Although it is methodologically impossible to explore and anticipate the space of all possible models of pitch processing and more sophisticated bottom-up mechanisms might theoretically suffice to explain the sweep pitch shift in sweeps and sweep trains, the feedback modulation mechanism introduced in this study is, to date, the only available account of the experimental data.

### Relation to predictive coding and hierarchical processing strategies

The presence of predictive feedback modulation in the subcortical sensory pathway has been shown before in humans [49, 50] and non-human mammals [51]. Previous studies often interpreted it in the context of the predictive coding framework [27, 28, 52], a theory of sensory processing that postulates that sensory information is encoded as prediction error; i.e., that neural activity at a given level of the processing hierarchy encodes the residuals of the sensory input with respect to a generative model encoded higher in the hierarchy.

The FM-feedback spectral model can also be understood in the light of this formalism: it presents three hierarchical layers of abstraction: the inputs from the peripheral system, the spectral layer, and the sweep layer. The top layer performs predictions on the sensory input incoming at the immediately lower representation of the hierarchy. However, unlike the classical predictive coding microcircuit where the generative model used to perform predictions and prediction error are kept in separate neural ensembles [53], the sweep network simultaneously holds a representation that is both, descriptive for its own representation and predictive for the immediately lower representation in the hierarchy.

Combining the generative model and stimulus representations in the same neural code solves some of the open questions of classical predictive coding architectures recently summarised by Denham and Winkler [54]: i) “what precisely is meant by prediction?”, ii) “which generative models [within the hierarchy] make the predictions?”, and iii) “what within the predictive framework is proposed to correlate with perceptual experience?”. In the FM-feedback spectral model, the predictions can be summarised as the probability distribution of patterns of activation expected to come next in the lower level given what has been encoded so far in the higher level. These conditional probability distributions are encoded in the feedback connections stemming from the neurons holding the high-level representation and targeting the neurons holding the lower level representations. Such connectivity patterns would represent the statistics between the representations in the two levels, and could have arisen naturally during development after sufficient exposure to the stimuli. The perceptual experience in the FM-feedback spectral model is encoded in the activation along the two hierarchical stages, which encode different aspects of the stimuli.

Another key difference between the FM-feedback spectral model’s architecture and the classical predictive coding microcircuit is that, rather than encoding the residuals of the spectral representation with respect to the FM-sweep representation, neurons in the spectral layer simply encode the spectral content of the stimulus. However, since the decoding of the predictable parts of the stimuli is faster, predictability potentially ensues a significant decrease on the amount of signal produced during the encoding. Such mechanism would explain why even expected stimuli, for which the residual should theoretically be zero, do still evoke measurable responses (e.g., [51]).

### Comparison with previous measurements of the sweep pitch shift

Our experimental findings qualitatively replicated the sweep pitch shift found in previous studies; namely, we found that the pitch elicited by FM-sweeps was biased towards the frequencies spanned in the ending part of the sweeps [17], and that the perceptual bias is monotonically related to the frequency span Δ*f* [18, 19]. On average, we estimated a putative linear relation between the pitch shift Δ*p* and Δ*f* of around *m* ≃ 0.38, slightly higher than Brady’s [17] (*m* ≃ 0.34 with transitions of 50 ms) and Nabelek’s [18] (*m* ≃ 0.32 with transitions of 40 ms) reports, and significantly higher than Rossi’s [19] (*m* = 1/6 ≃ 0.17 with transitions of 200 ms) estimation. Since Rossi’s transitions were 5 times longer than ours, the estimations are difficult to compare. However, the disagreement seems to indicate that the pitch shift is stronger with shorter durations. This observation would be fully compatible with the mechanism of predictive facilitation described in the FM-feedback spectral model: since the time to decode FM direction is independent of sweep duration, whilst only the most posterior part of the stimulus is facilitated in the short sweeps, in a long sweep the facilitation currents would affect a much larger portion of the sound, potentially including frequencies occurring even before $\bar{f}$.

In their original study Brady and colleagues [17] found that some sweeps of 20 ms duration elicited pitch values that coincided and even exceed the frequency span of the stimuli, especially in up sweeps. This perceptual extrapolation or *overshoot* has been replicated in two later studies [55, 56] and seems to further confirm the idea that the sweep pitch shift is driven by feedback between the sweep and spectral layer. However, the facilitation currents present in our model could not provoke activation of frequencies that are not initially present in the stimulus. One possibility is that the facilitation currents induce activation in neurons encoding frequencies that, although beyond the spectral range spanned by the sweep, are present in the stimulus spectrum. This scenario is unlikely in our data because we used frequency modulated sinusoids that elicit a unimodal distribution of responses in the auditory periphery. However, the three studies that showed the overshoot effect used spectrally rich stimuli. This would also explain why our model predicts smaller effect sizes than Brady’s data in Fig 9. To clarify this point further work could either test if, as predicted by our model, sinusoidal sweeps do not produce an overshoot, and if an extension of our model able to handle spectrally rich stimuli does reproduce the overshoot effect shown in previous experimental data.

The FM-feedback spectral model also provides for a mechanistic interpretation of the two additional experiments reported in Brady’s original study [17]. In Brady’s experiment II, the transient duration of the sweep is kept constant but its onset is varied across the stimulus duration. When the transient is located near the beginning of the stimulus, the greatest part of the sounds excites neurons encoding frequencies close to the posterior parts of the transient pushing the distribution of the responses away from the average frequency towards the ending frequencies of the sweep *f*_1_. This shift is larger than expected for a sound without a transient because of the feedback modulation of the later frequencies exerted by the sweep network. When the transient is located at the very end of the stimulus, the longer portion of the stimulus exciting *f*_0_ compensates for the shift in the frequency distribution, bringing the perceived pitch closer to the starting frequencies of the stimulus.

In Brady’s experiment III, the transient’s onset is kept constant and it is the duration of the transient that is varied. The decreased sweep pitch shift observed for shorter in comparison to longer transient durations can be explained by the FM-feedback spectral model as a consequence of the stimuli presenting a larger segment with the initial frequency, thus shifting the distribution of the responses towards *f*_0_.

### FM encoding and physiological location of the sweep and spectral layers

The earliest neural centre within the auditory pathway showing FM direction selectivity in mammals is the inferior colliculus [3–6], although thalamic nuclei (medial geniculate body) [6, 7] and auditory cortex [4, 8–11] show generally stronger DSIs. Thus, the sweep layer postulated in the FM-feedback spectral model could be implemented even at early stages of the auditory hierarchy. Similarly, since all the nodes in the ascending auditory pathway contain tonotopically arranged nuclei, the spectral layer could be putatively located as early as in the cochlear nucleus. The physiological location of the mechanisms described here remains an open question.

## Conclusion

In this work we have harnessed a well-established perceptual phenomenon to inform a model of FM direction encoding. We have shown that representative bottom-up models of pitch processing do not explain the pitch elicited by fast FM sweeps. Based on neurobiological considerations, we hypothesised that FM direction-selective neurons alter the way that spectral information is encoded via a feedback mechanism. We used the hypothesis to develop a model that proposes how this feedback modulation might be exerted and how it might affect the pitch percept elicited by FM sweeps. Although we cannot logically exclude other potential explanations for the effect, we provide evidence that our hypothesis is a likely and plausible mechanism underlying the encoding of formant transitions. These mechanisms could be part of a larger hierarchical network that transforms formant transitions into phonemes, phonemes into syllables, and syllables into words. Unravelling the fundamental building blocks of this hierarchy is a necessary prerequisite for a comprehensive understanding of the computational mechanisms underlying speech perception in the human brain.

## Materials and methods

### Ethics statement

The study was approved by the ethics committee of the University of Leipzig (ethics approval number 273/14-ff). All participants provided informed verbal consent.

### Measuring the sweep pitch shift in single sweeps

#### Participants

Eight participants (4 female), aged 22 to 31 (average 26.9) years old, were included in the study. They all had normal hearing thresholds between 250 Hz and 8 kHz (<25dB HL) according to pure tone audiometry (Micromate 304, Madsen Electronics). All reported at least five years of musical experience, but none of them was a professional musician. See the section Inclusion criteria bellow.

We considered a sample of *N* = 8 as sufficient for two reasons. First, the sweep pitch shift has been independently demonstrated in several previous studies [17–19]. The first experiment in our study is a replication of these previous studies that allowed us to quantify the sweep pitch shift. Second, low-level psychoacoustic phenomena are typically characterised by small inter-subject variabilities, so not many participants are necessary to demonstrate their generalisability [57]. We have selected 8 participants to ensure that that was the case, but the literature is populated with highly reproducible results that are inferred from experiments performed on populations as small as *N* = 4 [58]. Ours and previous data confirms that indeed the sweep pitch shift is present and shows the same direction and order of magnitude at the single subject level (S1 Fig; see also Tableau IV in [19] showing the effect in 18 subjects). Both experiments carried out in our study were taxing and extremely long, lasting for up to three hours per subject. Thus, while increasing the sample size would have not resulted in a stronger demonstration of the effect, it would have incurred an unjustified waste of resources.

#### Stimuli

Stimuli were 50 ms long frequency-modulated sweeps. Frequency was kept constant during the first and final 5 ms of the sweeps. The modulation was asymptotic (i.e., linear in the period *T* = 1/*f* space) and carried out in 40 ms. Stimuli were ramped-in and ramped-out with 5 ms Hanning windows overlapping the sections with constant frequency.

There were 30 single sweeps with 10 linearly distributed frequency spans Δ*f* ∈ [−600, 600] Hz and 3 average frequencies $\bar{f}\in \{900,1200,1500\}\;\text{Hz}$. For each sweep with a given Δ*f* and $\bar{f}$, the initial and final frequencies were $f_{0}=\bar{f}-\Delta f/2$ and $f_{1}=\bar{f}+\Delta f/2$.

Sounds were delivered by over-ear headphones Sennheiser (Sennheiser electronic GmbH & Co. KG; Germany) HD201 connected to a Realtek (Realtek Semiconductor Corp.; Taiwan) ALC1150 soundcard. Participants were required to adjust the loudness of the stimuli to a comfortable level during the pure-tone-test (see Inclusion Criteria), so that they had a wide range of pure tones to use as reference. The experiment was carried out in a quiet room. Stimuli were produced and delivered by a custom-made MATLAB (MathWorks, Natik, USA) script. Scripts running the experiment and generating the sounds are freely available in github.com/qtabs/fmPitch/experiment.

#### Experimental design

Each trial consisted of a sequential presentation of a target sweep and a probe pure tone. After the presentation, the participant was asked whether the second sound evokes a higher, equal, or lower pitch percept than the first sound. Participants were allowed to replay the sounds as many times as needed in case of doubt. After the response, the software adjusted the frequency of the probe tone by steps of ±*ϵ* = ±25 Hz, bringing the pitch of the sound closer to the participant’s percept (e.g., if the participant judged the target sweep as having a lower pitch than the probe tone, the frequency of the probe tone was reduced by 25 Hz). This procedure was repeated until the participant reported that the two sounds evoked the same pitch percept. Then, the frequency of the matched pure tone was stored as the perceived pitch of the sweep reported in that trial, and a new trial with a new target sweep began. The initial frequency of the probe tone was sampled from a Gaussian distribution centred on the average frequency $\bar{f}$ of the target sweep.

Each of the 30 sweeps was matched four times, so that there were 120 trials in total in the experiment. The relative order of the probe tone and the target sweep was reversed in half of the trials to assess if presentation order affects the sweep pitch shift. Thus, the experiment can be described as a 10 (10 different frequency spans) × 3 (3 average frequencies) × 2 (probe played first or last) factorial design.

#### Inclusion criteria

We initially recruited 22 participant candidates, which were screened by a first behavioural test assessing their capacity to match pure tones against pure tones (*pure-tone-test*), and then by a second behavioural test measuring their consistency when matching sweeps against pure tones (*sweep-test*). 14 of those 22 participants did not comply with the inclusion criteria: one was unable to match pure tones of the same frequency, 13 were unable to match sweeps against pure tones consistently. Consistency was assessed independently for each subject: i.e., the test did not evaluate whether the subject conformed to the results of other participants or if the subject showed a sweep pitch shift in any direction. The test only served to evaluate whether the subject approximately adjudicated the same pure tones to the same sweep, and was meant to exclude subjects that, due to poor pitch discrimination abilities or lack of motivation, were unable to perform the task.

The pure-tone-test was designed to ensure that participants had understood the task. We used the same experimental design as in the main experiment, including the same frequency step of 25 Hz, but both probe and target consisted of pure tones. During the pure-tone-test, the software provided feedback after each trial informing the participant whether the response was correct or incorrect. The pure-tone-test was divided in batches of six trials, and it concluded when the participant correctly matched the pitch of every trial in one batch. Most participants completed the pure-tone-test in the first batch; the participant excluded during the pure-tone-test failed to provide correct estimates for as many as six batches.

The *sweep-test* was used to evaluate whether participants could perform self-consistent judgements on the pitch of FM-sweeps. During the sweep-test, participants undertook a block of 12 trials consisting in 4 repetitions of the same 3 sweeps: $\left\{\Delta f=67\;\text{Hz},\bar{f}=900\;\text{Hz}\right\}$, $\left\{\Delta f=-200\;\text{Hz},\bar{f}=1200\;\text{Hz}\right\}$, and $\left\{\Delta f=-67\;\text{Hz},\bar{f}=1500\;\text{Hz}\right\}$. We chose these sweeps to ensure diversity of the sweep properties while keeping |Δ*f*| small enough to ensure that the sweeps would elicit an unequivocal pitch percept according to Hart’s law [59]. After the completion of this block, we scored the participant’s pitch matching consistency as the inverse of the average of the absolute differences between the reported pitch in each sweep. Participants with an average standard deviation larger than twice the frequency increment step 2*ϵ* = 50 Hz were excluded from the experiment.

Since Hart’s law [59] establishes that the sweeps used during the *sweep-test* elicit an unequivocal pitch percept, excluded participants were either unable or unwilling to perform consistent pitch judgements on sweeps. The inclusion of participant with inconsistent judgements would have contaminated the data with random guesses that could bias our estimations of the sweep pitch shift towards Δ*p* = 0. Six of the excluded participants reported no previous musical experience; the remaining 8 had at least five years of musical training.

#### Experimental procedure

The 8 included participants matched the remaining 27 sweeps in four additional blocks. No sweep type was repeated within a single block, and all sweeps were presented 4 times across the entire experiment, resulting in 27 trials per block. The order of the sweeps within each block was randomised and the relative position of the probe tone with respect to the target stimulus was pseudorandomised so that in half of the trials in each block the probe tone was presented before the target sweep. Participants were instructed to take rests between blocks and were allowed to take as many shorter rests between trials as needed. To encourage precision, a 5€ award was offered to participants that kept their self-consistency along the main experiment with the same criterium as in the evaluation: a smaller standard error than 2*ϵ* = 50 Hz within each sweep type. Only sweeps expected to yield the most unequivocal pitch sensation according to Hart’s law [59] (i.e., |Δ*f*| ≤ 200 Hz) were used to compute the overall self-consistency; participants were however unaware of this. Participants typically completed the experiment within 3 hours.

### Measuring the sweep pitch shift in sweep trains

#### Participants

The same 8 participants who completed the first experiment were invited to repeat the measurements with the new stimuli.

#### Stimuli

**Fig 12:**
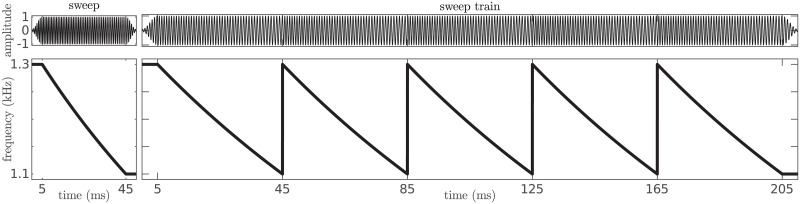
Examples of the stimuli. Waveform *s*(*t*) and instantaneous frequency *f*(*t*) of the sweep with $\bar{f}=1200\;\text{Hz}$ and Δ*f* = −200 Hz and its corresponding sweep train.

Stimuli were concatenations of 5 sweeps adding up to a total of 250 ms (sweep trains; see Fig 12). The sweeps were taken from a subset of 18 elements from the first experiment with 6 different frequency spans Δ*f* ∈ [−333, 333] Hz. To ensure continuity of the stimulus waveform, the sweeps were concatenated in the frequency domain (i.e., we computed the waveform of the stimuli by performing a Fourier transform over the concatenation of the time courses of the instantaneous frequencies). 5 ms Hanning windows were applied only at the very beginning and very end of the sweep trains.

#### Experimental design

The matching procedure was the same as in the first experiment: the participants matched the pitch of the sweep trains to probe pure tones whose frequency they could adjust with the aid of a computer software. To ensure that there were no effects of stimulus duration, the probe tones had the same duration as the sweep trains (i.e., 250 ms). As in the first experiment, each of the 18 sweep trains was matched four times, so that there were 72 trials in the second experiment. The relative order of the probe tone and the target sweep train was also reversed in half of the trials. Thus, the second experiment can be described as a 6 (different frequency spans) × 3 (average frequencies) × 2 (probe played first or last) factorial design.

#### Experimental procedure

Since the participants were already familiar with the task and proved to be able to match the pitch of FM-sweeps consistently, the experiment contained no pure-tone- or sweep- test. Four repetitions of the 18 sweep-trains were distributed across 5 blocks following the same principles as described in the first experiment. Participants typically completed the second experiment within 2 hours.

### Bottom-up models of pitch

#### Spectral models of pitch processing

The responses at the auditory nerve were computed with a model of the peripheral auditory system [22, 60]. The model’s output represents the expected firing rate *p*_*n*_(*t*) in a fibre of the auditory nerve associated with the *n*th cochlear channel (*n* = 1, 2, …, *N*) at an instant *t*. The frequency range of the cochlear model was discretised in *N* = 100 channels, spanning frequencies from *f*_min_ = 125 Hz to *f*_max_ = 10 kHz.

The perceived pitch corresponded to the expected cochlear channel *k*, *E*[*k*], according to a probability distribution *ρ* derived from the integral of *p*_*n*_(*t*) over the duration of the stimulus *L*:

$$\begin{array}{c} E\left[k\right]=\sum_{n}n\rho_{n}\;\text{with}\;\rho_{n}=\frac{\int_{0}^{L}dt\;p_{n}\left(t\right)}{\sum_{n}\int_{0}^{L}dt\;p_{n}\left(t\right)} \end{array}$$ (2)

To compare the predictions of the model with the experimental data, we also computed the expected channels *E*[*k*] associated to pure tones with the frequency of the average perceived pitch of each sweep.

#### Temporal models of pitch processing

The SACF used in this work follows the original formulation by Meddis and O’Mard [23, 24]. Essentially, this model poses the existence of an array of *M* periodicity detectors responding more saliently to a preferred period *δt*_*m*_. The instantaneous firing rate *A*_*m*_(*t*) of the *m*th periodicity detector (*m* = 1, 2, …, *M*) follows:

$$\begin{array}{c} \tau_{m}^{\text{SACF}}{\dot{A}}_{m}\left(t\right)=-A_{m}\left(t\right)+\sum_{n}p_{n}\left(t\right)\;p_{n}\left(t-\delta t_{m}\right) \end{array}$$ (3)

where the auditory nerve activity *p*_*n*_(*t*) in the cochlear channel *n* at an instant *t* is computed as in the previous section. The characteristic periods *δt*_*m*_ are uniformly distributed between *δt*_*m*_ = 0.5 ms and *δt*_*m*_ = 30 ms, which allows the model to capture periodicities corresponding to frequencies between 2 kHz and 135 Hz up to four lower harmonics. We kept a fixed integration constant $\tau_{m}^{\text{SACF}}=2.5\;\text{ms}$; using variable $\tau_{m}^{\text{SACF}}$ that depend linearly on *δt*_*m*_ (see details in [31, 61]) did not result in substantial changes in our results.

Stimuli presenting periodicities at a certain frequency *f* typically elicit peaks of activation in the detectors tuned to the preferred period *δt*_*m*_ = 1/*f* = *T*_0_ and to the periods corresponding to all subsequent lower harmonics *δt*_*m*_ = 2*T*_0_ = *T*_1_, *δt*_*m*_ = 3*T*_0_ = *T*_2_, etc. Thus, evidence for the period *T* at an instant *t*, *B*(*t*)_*T*_ can be represented as the $B{\left(t\right)}_{T}=\sum_{m\in \{M_{T}\}}A_{m}(t)$, where ${ℳ}_{T}$ are the indices of the periodicity detectors tuned to *T*, 2*T*, 3*T*, etc. (i.e., ${ℳ}_{T}=\{m:\delta t_{m}=nT\;\forall \;n\in [1,2,3,...]\}$). We estimated *B*(*t*)_*T*_ using four harmonics; extending or reducing the number of harmonics used to estimate *B*(*t*)_*T*_ did not significantly alter our results.

The perceived pitch corresponded to the expected period *T*, *E*[*T*], according to a probability distribution *ρ* derived from the integral of *B*_*T*_(*t*) over the duration of the stimulus *L*:

$$\begin{array}{c} E\left[T\right]=\sum_{T}T\rho_{T}\;\text{with}\;\rho_{T}=\frac{\int_{0}^{L}dt\;B_{T}\left(t\right)}{\sum_{n}\int_{0}^{L}dt\;B_{T}\left(t\right)} \end{array}$$ (4)

To compare the predictions of the model with the experimental data, we computed the expected period *E*[*T*] associated to pure tones with the frequency of the average perceived pitch of each sweep.

### Details on the predictive model of FM encoding

#### Spectral layer and pitch estimations

The spectral layer consists on an array of *N* = 100 neural populations that integrate the output of the peripheral model. Neural populations are modelled according to a mean-field derivation [62] on linear integrate-and-fire neurons that, although first formulated to describe dynamics in cortical regions dedicated to visual decision making, has shown a great versatility approximating the dynamics of many different cortical areas (e.g., [63]). We decided to use a simple point model without physiological detail because we do not know exactly the location in the brain of the system we are modelling.

The firing rate *h*_*n*_(*t*) of the *n*th ensemble follows the dynamics of a leaky integrator:

$$\begin{array}{c} \tau^{\text{pop}}\;{\dot{h}}_{n}^{f}\left(t\right)=-h_{n}^{f}\left(t\right)+\phi \left(I_{n}^{f}\left(t\right)\right) \end{array}$$ (5)

where *ϕ*(*x*) = (*cx* − *I*_0_)/(1 − *e*^−*g*(*cx* − *I*_0_)^) is the transfer function of the mean-field model and *τ*^pop^ are adaptive time constants:

$$\begin{array}{c} \tau_{e,i}^{\text{pop}}\left(h,I\right)=\tau_{e,i}^{\text{memb}}\;\Delta_{T}\frac{\partial_{x}{\phi \left(x\right)|}_{x=I}}{h} \end{array}$$ (6)

Δ_*T*_ = 1mV is the size of the spike initialisation of the neural model and $\tau_{e}^{\text{memb}}=20\;\text{ms}$ and $\tau_{i}^{\text{memb}}=10\;\text{ms}$ [44] are the neural membrane time constants for excitatory and inhibitory populations, respectively. Using adaptive integration time constants makes the populations to react faster to changes when they are marginally active and have weak synaptic inputs, a behaviour often reported in tightly connected populations of neurons [42]. This component is the key of the feedback mechanism used to increase the responsiveness of the populations encoding the expected parts of the sweeps (Fig 6). The analytic formulation of *τ*^pop^(*h*, *I*) stems from a theoretical study of networks of exponential-integrate-and-fire neurons [42].

Inputs $I_{n}^{f}(t)$ were modelled with AMPA synaptic dynamics [41]. AMPA synapses present short time constants that are able to preserve the fine temporal structure of auditory input, and thus are the major receptor type conveying bottom-up communication in the auditory pathway (e.g., [64]).

$$\begin{array}{c} I_{n}^{f}\left(t\right)=J_{\text{in}}^{\text{AMPA}}\sum_{k}\omega_{nk}^{\text{in}}S_{\text{in},k}^{\text{AMPA}}\left(t\right) \end{array}$$ (7)

where $J_{\text{in}}^{\text{AMPA}}$ is the effective synaptic efficacy of the peripheral input. We allowed some dispersion in the propagation from the peripheral model to the spectral layer by using a Gaussian-shaped connectivity matrix. This ensured that the bandwidth of the self-excitation in the spectral representation is independent of the number of cochlear channels:

$$\begin{array}{c} \omega_{nm}^{\text{in}}=\frac{1}{\sqrt{\sigma_{\text{in}}}}e^{-\frac{{\left(m-n\right)}^{2}}{2\sigma^{\text{in}}}} \end{array}$$ (8)

where the normalisation factor $\sqrt{\sigma_{\text{in}}}$ ensures that the total input to a population under a uniform peripheral input remains the same regardless of the dispersion *σ*_in_. The synaptic gating variable $S_{\text{in},n}^{\text{AMPA}}(t)$ follows [41]:

$$\begin{array}{c} \tau^{\text{AMPA}}{\dot{S}}_{\text{in},n}^{\text{AMPA}}\left(t\right)=-S_{\text{in},n}^{\text{AMPA}}\left(t\right)+p_{n}\left(t\right) \end{array}$$ (9)

Note that we used the index *^f^* to denote variables in the spectral layer. The perceived pitch corresponded to the expected cochlear channel *k*, *E*[*k*], according to a probability distribution *ρ* derived from the integral of $h_{n}^{f}(t)$ over the duration of the stimulus *L* (cf. Eq (2)):

$$\begin{array}{c} E\left[k\right]=\sum_{n}n\rho_{n}\;\text{with}\;\rho_{n}=\frac{\int_{0}^{L}dt\;h_{n}^{f}\left(t\right)}{\sum_{n}\int_{0}^{L}dt\;h_{n}^{f}\left(t\right)} \end{array}$$ (10)

The time constant *τ*^AMPA^ = 2 ms was taken from the literature [41]. The effective conductivity $J_{\text{in}}^{\text{AMPA}}=0.38\;\text{nA}$ was manually tuned within the realistic range such that the peripheral system would elicit firing rates on the range 5Hz ≤ *h*_*n*_(*t*) ≤ 100 Hz in the integrator ensembles. The transfer function and its parameters, empirically derived for networks of integrate-and-fire neurons, were taken from [62].

#### Sweep layer and direction selectivity

We used delayed excitation [10, 26, 35] to model FM-direction selectivity. Two additional mechanisms for FM direction selectivity have been identified in IC, MGB and auditory cortex in the animal electrophysiology literature: asymmetric sideband inhibition [3, 35, 36], and duration sensitivity [6, 36, 37]. Although both delayed excitation and sideband inhibition contribute to direction selectivity in the mammal auditory pathway [3, 35, 36], the two mechanisms are often redundant and yield equivalent results when embedded in a neuronal model [14]. We chose to use delayed excitation alone for simplicity but, given that all models show similar direction and rate selectivity to FM-sweeps, replacing it by or adding any extra mechanism is unlikely to affect the model predictions.

The sweep layer consists of four arrays of *N* = 100 neural populations following the same dynamics described in the previous section (i.e., Eq (5)). From the four arrays, two (one excitatory, one inhibitory) are tuned to up sweeps, and two (again, one excitatory and one inhibitory) are tuned to down sweeps (Fig 3). The neural populations are characterised by the instantaneous firing rates $h_{n}^{↑e}(t),h_{n}^{↑i}(t)$ (*up*) and $h_{n}^{↓e}(t),h_{n}^{↓i}(t)$ (*down*), and receive synaptic inputs $I_{n}^{↑e}(t),I_{n}^{↑i}(t)$ (*up*) and $I_{n}^{↓e}(t),I_{n}^{↓i}(t)$ (*down*), respectively for excitatory and inhibitory populations. Although the transfer functions *ϕ*(*x*) are the same for all the ensembles, the parameters *c*, *I*_0_, and *g* are different for excitatory and inhibitory populations [62].

Excitatory and inhibitory inputs to populations in the *sweep layer* are modelled according to AMPA-like and GABA-like synaptic gating dynamics [41]:

$$\begin{array}{ccc} {\dot{S}}_{\alpha ,n}^{\text{AMPA}}\left(t\right) & = & -\frac{S_{\alpha ,n}^{\text{AMPA}}\left(t\right)}{\tau^{\text{AMPA}}}+h_{n}^{\alpha e}\left(t\right)+\sigma \xi ,\;\alpha =↑,↓,f\\ {\dot{S}}_{\alpha ,n}^{\text{GABA}}\left(t\right) & = & -\frac{S_{\alpha ,n}^{\text{GABA}}\left(t\right)}{\tau^{\text{GABA}}}+h_{n}^{\alpha i}\left(t\right)+\sigma \xi ,\;\alpha =↑,↓ \end{array}$$

where *ξ* is an uncorrelated Gaussian noise sampled independently for each synapse and instant *t*, and *σ* = 0.0007 nA is the amplitude of the noise [62]. The total synaptic input for each population is then:

$$\begin{array}{ccc} I_{n}^{↑e}\left(t\right) & = & J_{f}^{\text{AMPA}}\sum_{m}\omega_{nm}^{f↑}S_{f,m}^{\text{AMPA}}\left(t-\delta t_{nm}\right)-\\ & & J^{\text{GABA}}(\sum_{m}\omega_{nm}^{ie}S_{↓,m}^{\text{GABA}}\left(t\right)+S_{↑,n}^{\text{GABA}}\left(t\right))+I_{\text{bkg}}^{E}\\ I_{n}^{↑i}\left(t\right) & = & J_{s}^{\text{AMPA}}\sum_{m}\omega_{nm}^{ei}S_{↑,m}^{\text{AMPA}}\left(t\right)+I_{\text{bkg}}^{I}\\ I_{n}^{↓e}\left(t\right) & = & J_{f}^{\text{AMPA}}\sum_{m}\omega_{nm}^{f↓}S_{f,m}^{\text{AMPA}}\left(t-\delta t_{nm}\right)-\\ & & J^{\text{GABA}}(\sum_{m}\omega_{nm}^{ie}S_{↑,m}^{\text{GABA}}\left(t\right)+S_{↓,n}^{\text{GABA}}\left(t\right))+I_{\text{bkg}}^{E}\\ I_{n}^{↓i}\left(t\right) & = & J_{s}^{\text{AMPA}}\sum_{m}\omega_{nm}^{ei}S_{↓,m}^{\text{AMPA}}\left(t\right)+I_{\text{bkg}}^{I} \end{array}$$

where $I_{\text{bkg}}^{E}$ and $I_{\text{bkg}}^{I}$ are constant background inputs putatively sourced in external neural populations [62].

The excitatory-to-inhibitory and inhibitory-to-excitatory connectivity matrices *ω*^*ei*^ and *ω*^*ie*^ are Gaussian shaped and centred in the identity matrix:

$$\begin{array}{c} \omega_{nm}^{\alpha }=e^{-\frac{{\left(n-m\right)}^{2}}{2\sigma_{\alpha }}},\;\alpha =ei,ie \end{array}$$ (11)

The remaining connectivity matrices *ω*^*f*↑^ and *ω*^*f*↓^ are defined to constraint the up (down) feed to inputs from lower (higher) frequencies and to limit the range of the connection to a small number of populations Δ_*ωf*_ of the spectral representation:

$$\begin{array}{ccc} \omega_{nm}^{f↑} & = & \left\{ \begin{array}{cc} 1 & \;\text{if}\;\;0\leq n-m\leq \Delta_{\omega f}\\ 0 & \;\text{otherwise} \end{array}\right.\\ \omega_{nm}^{f↓} & = & \left\{ \begin{array}{cc} 1 & \;\text{if}\;\;0\leq m-n\leq \Delta_{\omega f}\\ 0 & \text{otherwise} \end{array}\right. \end{array}$$

The free parameters were initialised to standard values (the effective conductivities $J_{f}^{\text{AMPA}}$, *J*^GABA^, and $J_{s}^{\text{AMPA}}$, according to [62]; the baseline delay *δt*_0_ to 2 ms/channel; and the dispersion constants *σ*_in_, *σ*_ei_, *σ*_ei_, and Δ_*ωf*_, to 0.1*N*) and manually tuned so that the networks showed direction selectivity for the FM-sweep characteristics (duration, rates, Δ*f*) of the stimuli used in the first part of the study. Unless stated otherwise, all simulations listed in this work correspond to the parameters listed in Table 1.

**Table 1:** Model parameters. Most parameters were taken from the original studies that derived the mean field approximations used in the model and are cited accordingly. Other free parameters, like the number of bins of the tonotopic axis *N*, were fixed to reasonable but arbitrary values at the beginning of the model construction and were not adjusted during the analyses (*ad-hoc*). Free parameters that were manually tuned are labelled as *tuned (x)*, where *x* is: 1, for parameters tuned so that the spectral layer integrates the peripheral representation correctly; 2, for parameters tuned to achieve FM-direction selectivity; and 3, for parameters tuned so that the feedback signalling resulted in a fair fit between the model’s pitch predictions and the experimental observations. Short description of the parameters in the last column are further explained along the Methods section. Connectivity parameters are more eloquently described in Fig 13.

parameter	value (unit)	source	description
*N*	100 channels	ad-hoc	number of cochlear channels
*dt*	0.1 ms	ad-hoc	time step for the numerical integration
periph *dt*	0.01 ms	ad-hoc	*dt* in the peripheral model
periph *f*_min_	125 Hz	[60]	best frequency of the first cochlear channel
periph *f*_max_	10000 Hz	[60]	best frequency of the last cochlear channel
$\tau_{e}^{\text{memb}}$	20 ms	[44]	membrane time constant of excitatory neurons
$\tau_{i}^{\text{memb}}$	10 ms	[44]	membrane time constant of inhibitory neurons
Δ	1 mV	[42]	action potential initiation sharpness
*c*^excitatory^	310 (V nC)^−1^	[62]	transfer function parameters (excitatory populations)
$I_{0}^{\text{excitatory}}$	125 Hz	[62]	transfer function parameters (excitatory populations)
*g*^excitatory^	0.16 s	[62]	transfer function parameters (excitatory populations)
*c*^inhibitory^	615 (V nC)^−1^	[62]	transfer function parameters (inhibitory populations)
$I_{0}^{\text{inhibitory}}$	177 Hz	[62]	transfer function parameters (inhibitory populations)
*g*^inhibitory^	0.087 s	[62]	transfer function parameters (inhibitory populations)
$I_{\text{bkg}}^{E}$	0.23 nA	[62]	baseline input current (excitatory populations)
$I_{\text{bkg}}^{I}$	0.10 nA	[62]	baseline input current (inhibitory populations)
*σ*	0.0007 nA	[62]	synaptic noise amplitude
*γ*	0.641	[41]	NMDA coupling factor
*τ*^AMPA^	2 ms	[41]	decay time constant of AMPA synapses
*τ*^GABA^	5 ms	[41]	decay time constant of GABA synapses
*τ*^NMDA^	100 ms	[41]	decay time constant of NMDA synapses
$J_{\text{in}}^{\text{AMPA}}$	0.38 nC	tuned (1)	AMPA conductivity of the peripheral input
$J_{f}^{\text{AMPA}}$	0.55 nC	tuned (2)	AMPA conductivity of the spectral populations
$J_{s}^{\text{AMPA}}$	0.67 nC	tuned (2)	AMPA conductivity of the sweep populations
*J*^GABA^	0.30 nC	tuned (2)	GABA conductivity (sweep populations only)
*J*^NMDA^	0.05 nC	tuned (3)	NMDA conductivity (feedback connections only)
*σ*_in_	0.1*N* channels	tuned (2)	dispersion in peripheral input to spectral populations
*σ*_ie_	0.5*N* channels	tuned (2)	dispersion in inhibitory-to-excitatory connections
*σ*_ei_	0.03*N* channels	tuned (2)	dispersion in excitatory-to-inhibitory connections
Δ*t*_0_	1 ms/channel	tuned (2)	baseline delay of the delayed-excitation mechanism
Δ_*ωf*_	0.05*N* channels	tuned (2)	connection width (spectral to sweep populations)
Δ_*ωs*_	0.05*N* channels	tuned (3)	connection width of the feedback (sweep to spectral)
*w*_*ωs*_	0.05*N* channels	tuned (3)	connection gap of the feedback (sweep to spectral)

The direction selectivity index (DSI; e.g., [11]) described in the Results section was computed as the proportion of the activity elicited in a network by an up sweep minus the activity elicited in the same network by a down sweep with the same duration and frequency span:

$$\begin{array}{c} {\text{DSI}}^{\alpha }=\frac{\sum_{n}\int \;dt({\left[h_{n}^{\alpha e}\left(t\right)\right]}_{+\Delta f}-{\left[h_{n}^{\alpha e}\left(t\right)\right]}_{-\Delta f})}{\sum_{n}\int \;dt({\left[h_{n}^{\alpha e}\left(t\right)\right]}_{+\Delta f}+{\left[h_{n}^{\alpha e}\left(t\right)\right]}_{-\Delta f})}\;\alpha =↑,↓ \end{array}$$ (12)

where ${\left[h_{n}^{\alpha e}(t)\right]}_{\Delta f}$ is the firing rate $h_{n}^{\alpha e}(t)$ elicited in the network by a sweep with a frequency span Δ*f*.

#### Feedback connections

Feedback connections from the sweep layers to the spectral layer were modelled according to NMDA-like synaptic gating dynamics with a finite rising time constant [41].

$$\begin{array}{ccc} {\dot{S}}_{\alpha ,n}^{\text{NMDA}}\left(t\right) & = & -\frac{S_{\alpha ,n}^{\text{NMDA}}\left(t\right)}{\tau^{\text{NMDA}}}+\sigma \xi \\ & & +(1-S_{\alpha ,n}^{\text{NMDA}}\left(t\right))\gamma \;h_{n}^{\alpha e}\left(t\right),\;\alpha =↑,↓ \end{array}$$

with *γ* = 0.641. NMDA currents are added to the total synaptic input of the neurons in the spectral layer as an additional term in (7):

$$\begin{array}{c} I_{n}^{f}\left(t\right)\to {\hat{I}}_{n}^{f}\left(t\right)=I_{n}^{f}\left(t\right)+J^{\text{NMDA}}\sum_{\alpha =↑,↓}\sum_{m}\omega_{nm}^{\alpha f}S_{\alpha ,m}^{\text{NMDA}}\left(t\right) \end{array}$$

**Fig 13:**
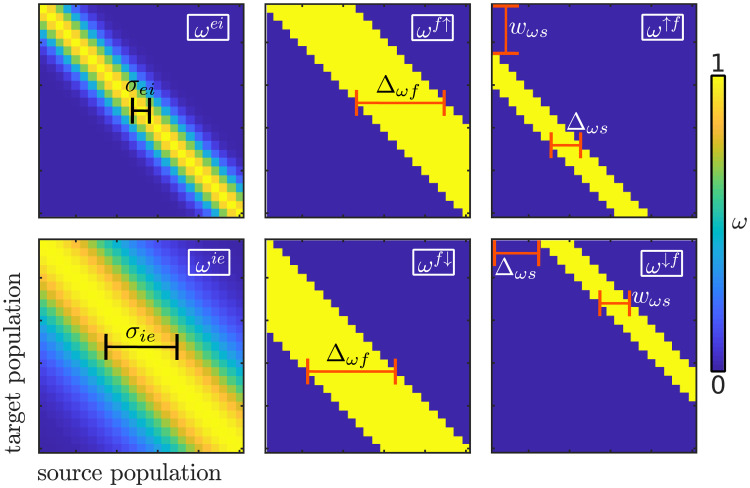
Connectivity matrices. Matrices show the connection between the first 25 ensembles of each source-target group. From left to right, matrices correspond to: excitatory-to-inhibitory *ω*^*ei*^, inhibitory-to-excitatory *ω*^*ie*^; bottom-up AMPA connections spectral-to-up *ω*^*f*↑^, spectral-to-down *ω*^*f*↑^; and feedback NMDA connections up-to-spectral *ω*^↑*f*^, down-to-spectral *ω*^↓^ *f*. Labels are encircled in a white square in the top right of each plot. The free parameters of each connectivity matrix are defined geometrically in the plots.

The connectivity matrices $\omega_{nm}^{\alpha ↑}$, $\omega_{nm}^{\alpha ↓}$ were chosen such that the target of the NMDA-driven activation was limited to a number of Δ_*ωs*_ and leave a gap of *w*_*ωs*_ populations between the centre frequency of the source and target ensembles (see Fig 13, right):

$$\begin{array}{ccc} \omega_{nm}^{↑f} & = & \left\{ \begin{array}{cc} 1 & \;\text{if}\;\;w_{\omega s}\leq m-n\leq \Delta_{\omega s}+w_{\omega s}\\ 0 & \;\text{otherwise} \end{array}\right.\\ \omega_{nm}^{↓f} & = & \left\{ \begin{array}{cc} 1 & \;\text{if}\;\;w_{\omega s}\leq n-m\leq \Delta_{\omega s}+w_{\omega s}\\ 0 & \text{otherwise} \end{array}\right. \end{array}$$

The gap *w*_*ωs*_ > 0 is enforced to avoid resonances between sweep-selective and spectral populations with the same centre frequency during the encoding of pure tones. The free parameters were initialised to standard values (the NMDA conductivity *J*^NMDA^ to the value recommended by [62], and the connectivity parameters *w*_*ωs*_ and Δ_*ωs*_ to 0.1*N*) and manually tuned so that the pitch predictions of the model (as computed in Eq (10)) matched the empirical data.

## Supporting information

S1 FigSubject specific estimations of the linear fits between the pitch shift Δ*p* and Δ*f*.Each plot shows the slopes *m* of the linear fit $f_{\text{perceived}}∼\bar{f}+m\;\Delta f$ for each subjects, for the single sweep (left) and sweep train (right) stimuli; error bars mark the 95% confidence intervals of the estimations.(EPS)

S2 FigEffect of the presentation order on Δ*p*.Kernel density estimations of the difference between the perceived pitch evaluated when the sweep was presented before the probe tone $f_{\text{perceived}}^{\leftarrow }$ and the perceived pitch evaluated when the probe tone was presented before the sweep $f_{\text{perceived}}^{\to }$; no systematic effect of the presentation order was found for any of the conditions. Each sample of the distributions corresponds to the difference of the average perceived pitch between presentation orders of the same Δ*f* for a given subject and centre frequency (*N* = 8 × 3 = 24). Error bars show the average and the standard error of the groups. Average difference across Δ*f* was 145Hz ± 227Hz, largely overlapping 0.(EPS)

S1 TableSummary statistics on the relationship between the perceived pitch and the frequency span for single sweeps and sweep trains.The slope of the linear fit, Pearson’s correlation *r*_*p*_, and Spearman’s correlation *r*_*s*_ for the relationship between *f*_perceived_ and Δ*f* are presented for each centre frequency $\bar{f}$ and direction of the presentation (probe before sweep, →; and sweep before probe, ←). Spearman’s correlation is systematically larger than Pearson’s, indicating that the elicited pitch is related to Δ*f* in a non-linear monotonic way.(TEX)

S1 SoundsStimuli used in the experiments.Each waveform corresponds to each of the single-sweeps and sweep-trains used in the first and second experiment, respectively. File names indicate the properties of the stimulus as follows: [sweep/train]_fbar< $\bar{f}$ >_delta<Δ*f* >.wav; e.g., train_fbar1200Hz_delta-333Hz.wav is the sweep-train with $\bar{f}=1200\;\text{Hz}$ and Δ*f* = −333Hz used in the second experiment.(ZIP)
